# The utility of the Historical Clinical Risk -20 Scale as a predictor of outcomes in decisions to transfer patients from high to lower levels of security-A UK perspective

**DOI:** 10.1186/1471-244X-10-76

**Published:** 2010-09-29

**Authors:** Mairead Dolan, Regine Blattner

**Affiliations:** 1 Centre for Forensic Behavioural Science, Monash University and the Victorian Institute for Forensic Mental Health, 505 Hoddle Street, Clifton Hill, Victoria, 3068, Australia; 2 Department of Psychiatry, Laureate House, Wythenshawe Hospital, Southmoor Road, Manchester M23 9LT

## Abstract

**Background:**

Structured Professional Judgment (SPJ) approaches to violence risk assessment are increasingly being adopted into clinical practice in international forensic settings. The aim of this study was to examine the predictive validity of the Historical Clinical Risk -20 (HCR-20) violence risk assessment scale for outcome following transfers from high to medium security in a United Kingdom setting.

**Methods:**

The sample was predominately male and mentally ill and the majority of cases were detained under the criminal section of the Mental Health Act (1986). The HCR-20 was rated based on detailed case file information on 72 cases transferred from high to medium security. Outcomes were examined, independent of risk score, and cases were classed as "success or failure" based on established criteria.

**Results:**

The mean length of follow up was 6 years. The total HCR-20 score was a robust predictor of failure at lower levels of security and return to high security. The Clinical and Risk management items contributed most to predictive accuracy.

**Conclusions:**

Although the HCR-20 was designed as a violence risk prediction tool our findings suggest it has potential utility in decisions to transfer patients from high to lower levels of security.

## Background

Over the last 3 decades there have been significant developments in the field of violence risk assessment and management. It is increasingly recognized that individuals with mental disorder have an increased (4 to 6 times higher) risk of committing a violent crime [1,2]. Since the work of Monahan [3] unstructured clinical approaches to risk assessment in psychiatric patients have been questioned due to their low levels of accuracy. The literature suggests that there are a number of factors that are associated with violence and poor outcome in patients discharged from civil and forensic settings including major mental illness, substance abuse and psychopathy [4-7]. Over the last 15 years there have been notable developments in systematizing the risk assessment field which have led to the introduction of a number of risk assessment tools that provide a more structured approach to decision making [6,8,9]. The latter Structured Professional Judgment (SPJ) approach provides guidelines for assessing risk using systematized, empirically based, risk factors that can be coded but can still allow flexibility to take account of case-specific issues. One of the most researched instruments to use a SPJ approach is the Historical Clinical Risk-20 scale [8-10]. This measure has 10 historical, relatively static factors that do not change over time, and 10 dynamic (5 clinical and 5 risk management) items that are subject to change with treatment. See table 1 for item content. There are now a substantial number of international studies looking at the validity of the HCR-20 as a violence risk assessment tool. These include studies from Canada, Sweden, the Netherlands, Scotland, Germany, England and the United States. See [6,9-13]. Most of the published studies have focused on the validity of measures such as the HCR-20 in predicting in-patient and post discharge violence and aggression in male samples, although there is increasing data on female patients [14,15].

**Table 1:** HCR-20 item content

Historical Items	
H1	Previous Violence

H2	Young Age at First Violent Incident

H3	Relationship Instability

H4	Employment Problems

H5	Substance Use Problems

H6	Major Mental Illness

H7	Psychopathy

H8	Early maladjustment

H9	Personality Disorder

H10	Prior Supervision Failure

	

**Clinical Items**	

C1	Lack of Insight

C2	Negative Attitudes

C3	Active Symptoms of Major Mental Illness

C4	Impulsivity

C5	Unresponsive to Treatment

	

**Risk Management Items**	

R1	Plans Lack Feasibility

R2	Exposure to Destabilizers

R3	Lack of Personal Support

R4	Noncompliance with Redemption Attempts

R5	Stress

Interestingly, we previously [16] looked at the predictive validity and clinical utility of the HCR-20 as a predictor of more generic post discharge outcome in patients discharged from medium secure care to the community in the UK. We found that the HCR-20 was a good predictor of self-reported violence, readmission, and particularly readmission under the criminal sections of the England and Wales Mental Health Act, 1986, but did not necessarily relate to the intensity of supervision post discharge. This suggested that the HCR-20 may be a useful instrument for assessing the risk of poor outcome (in more general terms than violent recidivism) in decisions to transfer patients from higher to lower levels of security including the community. This led us to wonder if this instrument had value in predicting outcome decisions across levels of security in the forensic rehabilitation process.

In England and Wales (E&W) and most European and Canadian and United States (US) forensic services, the rehabilitation of high security patients who are detained in High Security Psychiatric Hospitals (HSPHs) usually occurs via transfer to progressively lower levels of security prior to discharge into the community [17,18]. Apart from the UK few jurisdictions have systematically looked at the outcomes of patients across levels of security and international comparative data is currently quite limited. A review of the medium to long term outcomes of discharges from HSPHs in E&W, with follow up between 2-11 years, suggests that hospital readmission rates range between 7 - 22% [19]. Reconviction studies of released HSPH patients also suggest that the rate of serious reconvictions ranges from 3% to 24% overall, [20-22]. However, Davison et al. [23] reported that rates were notably higher in patients with a diagnosis of Axis II personality disorder rather than an Axis I disorder.

A range of independent clinical studies suggest that poor outcome for HSPH patients appears to be linked with a variety of risk factors including; younger age, a higher number of previous convictions, a history of psychiatric admissions, mental impairment, psychopathy or a sexual index offence [19,24-26], but few of these risk factors have been examined together in the context of a comprehensive risk assessment protocol. Given that SPJ approaches to risk assessment have been adopted as good clinical practice in most US and European jurisdictions, but there is limited evidence on the applicability in clinical practice, we wanted to investigate the utility of the HCR-20 in decision making on transfers from high to medium and lower levels of security in a UK context.

Available data from the limited number of studies examining the outcomes of HSPH patients transferred to medium security in E&W suggest that between 26-33% are returned to high security, and between 9-11% are reconvicted for serious offences [26-28]. Given the growing interest in the use of more structured clinical risk assessment and management tools in clinical decision making [6,9,29-35], we investigated the potential utility of a Structured Professional Judgment (SPJ) approach to violence risk assessment using the Historical Clinical Risk violence risk scheme (HCR-20; [8]) in the decision to transfer cases between high and lower levels of forensic secure care. The HCR-20 has repeatedly been shown to be a robust predictor of institutional and community violence in mentally disordered samples across a range of settings and international centers [9,16,33-39].

We have previously shown that the HCR-20 was actually a useful predictor of self-reported violence and readmission to hospital in patients transferred from medium and low secure care to the community [16] and that clinically based supervision levels post discharge was unrelated to systematic risk assessment status [16]. As there was one report that suggested that the HCR-20 was useful in characterizing risk status in patients managed by community mentally health services in the UK [40], we examined its utility as an assessment tool in decisions to transfer patients from high to lower levels of security.

## Methods

### Study participants

The study was conducted in the Edenfield Centre Medium secure unit in the North West region of E&W. The 2005-6 cohort under study was based on all HSPH patients admitted to the Edenfield medium secure unit (MSU) psychiatric facility from its inception in September 1986 to June 2001, and who had a terminated MSU admission episode by May 2002. That is, they had been discharged to the community or returned to the HSPH from the MSU by May 2002. In cases where a patient had several admissions to the MSU, the first admission was used as the index admission case for the purposes of this study. The study criteria generated a total of 72 consecutive patients discharged from HSPH to the Edenfield Centre whose index admission to the latter unit had terminated either through discharge to the community or lower levels of security (success), or transfer back to high security/reconviction (failure). Of all admissions to the Edenfield centre, this HSPH sample represented 11% of all admissions to the unit during that time period. The remainder of the transfers/admissions had come from prisons or from area/local mental health services. The majority were detained under section 41 (restriction order) of the UK Mental Health Act 1986. That is, the patients were detained in hospital following a court appearance for an offence that was deemed associated with mental disorder requiring inpatient treatment and whose discharge could only be approved by the Home Office (now Ministry of Justice) or following appeal to a Mental Health Review Tribunal.

The mean age of the HSPH cohort under study was 36.4 years (SD = 11.5). Sixty- three (87%) were male and 57 (79%) were Caucasians. The remainder were of Afro-Caribbean (10%) or Asian/mixed race origin (11%). Clinical case files, which record multi-axial diagnoses, indicated that the majority had an Axis I clinical diagnosis particularly schizophrenia, but there were high rates of co-morbidity with Axis II pathology. A significant proportion of the cohort met criteria for substance abuse dependence. Forty-seven patients (65%) had more than one clinical diagnosis recorded. See table 2.

**Table 2:** Clinical diagnosis according to DSM-IV (several diagnoses possible, n = 72)

Organic brain syndromes	6 (8%)
Schizophrenia or -related disorders	48 (67%)
Affective disorders	4 (6%)
Alcohol-related disorders (misuse or dependency)	22 (31%)
Substance-related disorders (misuse or dependency)	22 (31%)
Personality disorder	22 (31%)
Neurotic disorders	3 (4%)
Mental Impairment	6 (8%)
Co morbidity between disorders	47 (65%)

The majority (55, 76%) had previous admissions to a psychiatric hospital. Fifty-nine (82%) had previous convictions with a range of 1-35 offences. The mean age at first conviction was 19.5 years (SD = 8.3). The frequency of particular index offences were as follows; violence against others (64%); violent sex offences (17%); arson with intent to endanger life and criminal damage (19%). See table 3.

**Table 3:** Index offences (index offences not mutually exclusive, n = 72)

Offences against person	
murder/manslaughter	23 (32%)
attempted murder/serious wounding	23 (32%)
Sexual offences	
Rape	7 (9.7%)
against children/teenagers	3 (4.1%)
Other sexual offence	3 (4.1%)

Offences against property	
Arson	14 (19.4%)
robbery/burglary	10 (13.8%)

Other offences	9 (12.5%)
No offence	0 (0%)
Several offences n (%)	19 (26.3%)

Other offences include: criminal damage, breach of peace, severely disorderly behaviour, kidnapping, possessing weapons or imitation firearms with intent, driving without licence and taking conveyance.

Prior to transfer to the MSU, the mean length of stay at the HSPH was 7.4 years (SD = 5.8). The majority (59, 82%) were transferred to the MSU on trial leave to test their suitability for rehabilitation into the community. The mean length of MSU stay was 1.2 years (SD = 1.0).

### Procedure

The Local Research and Ethics Committee (LREC) granted approval for the study. Responsible Medical Officers (RMOs) gave consent for access to patient's files.

The HCR-20 was rated from the detailed case files based by a trained psychiatrist on the data available in the medium secure unit following transfer from high security. The case files were reviewed and the HCR-20 scored based on data available prior to their transfer out of, or discharge from, the medium secure unit, but this was conducted blind to subsequent outcomes. The HCR-20 scale has ten Historical-H items, five Clinical-C items, and five Risk-R items. The H items are based on empirical literature on violence risk assessment and tend to remain static over time. The C and R items are amenable to change with intervention and supervision. All 20 items are coded using a "0" rating for absence of an item, "1" for possible presence of the item and "2" for definite evidence for this item. Descriptors and criteria for each item are provided in the manual [8] but HCR-20 items are listed in table 1.

### Outcome data

Outcome was classed as "success" or "failure" based on the work of Quinn and Ward [27] and Cope and Ward [28] who used similar criteria for outcome measures in their study. Success was based on successful rehabilitation from the MSU to the community with no adverse events (readmission/reconviction) during the study period.

Failure was based on:

(i) Direct return to the HSPH,

(ii) Return to the HSPH after discharge to the community and

(iii) Reconviction for a serious offence after discharge to the community. Re-conviction data was extracted from combined sources including case files and the official records in the Offenders Index of the Home Office. A reconviction was regarded as being "serious" in cases of murder, manslaughter, assault, rape, indecent assault towards adult male, adult female or child, robbery and arson, based on the criteria of Bailey and MacCulloch [22].

### Data analysis

Data were analyzed using the Statistical Package for Social Sciences SPSS for Windows (version 14) Chicago Illinois Inc. Where possible, outcome data was coded into dichotomous groups e.g. outcome present or absent. Receiver Operating Characteristics (ROC) analyses [28], were used to examine the predictive validity of the HCR-20 score for dichotomous outcome measures as they are relatively independent of the base rate for violence in a given population. ROCs also offer the advantage of plotting the trade-off between sensitivity (true positive rate) and 1-specificity (false positive rate). The Area under the curve (AUC) statistic ranges from 0 (perfect negative prediction) to 1 (perfect positive prediction) with 0.50 representing a chance level of prediction. ROC AUC statistics of 0.76 approximate to Cohen's d of 1 which is considered a large effect size [7,38].

## Results

### General outcome

Overall, 32 patients (44.4%) were rated as having a successful outcome in that they were successfully rehabilitated to the community with no adverse events during the study period.

Forty patients (55.5%) had an outcome that was classed as a "failure" based on the assigned categories. Thirty-three (46%) patients returned directly to the high-security hospital from the MSU; one patient was recalled to the HSPH with treatment-resistant mental illness; one patient was recalled after a serious re-conviction and five further patients were re-convicted of serious offences.

### Reconviction data- Community outcomes

Of the 39 patients (54%) who were discharged to the community (mean 6 years SD 3.6), 8 (21%) were reconvicted. Mean length of time until re-offending was 5.25 years (SD = 3.7). Six (15%) were for serious offences (violence against the person).

### The predictive validity of the HCR-20 for outcomes

**Figure 1:**
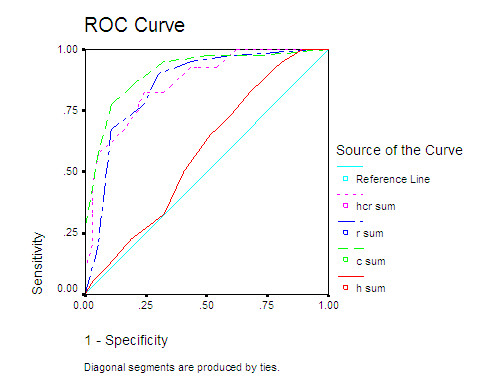
**Area under curve: Historical, clinical and risk subscale as well as total HCR-20 score as a predictor of the outcome "failure"**.

The mean total HCR-20 score was 22.06 (SD 7.2), The H score was 12.47 (SD 3.5), C was 4.29 (SD 3.0) and R 5.29 (SD2.5). Table 3 shows the ROC curve analyses for the total and subscale scores of the HCR-20 for "failed outcome". The HCR-20 total score was a reasonably robust predictor of "failure". Analysis of the subscale scores indicated that the C and R subscales rather than the H subscale were significantly better than chance predictors. See Table 4 and figure 1.

**Table 4:** HCR-20 subscale and total HCR-20 score as predictor for outcome "failure"

HCR-20 subscales	Area under the curve (AUC)	Std error	Significance	95% CI Lower	95% CI Upper
Historical	0.59	0.069	0.16	0.46	0.71
Clinical	0.907	0.035	0.00	0.839	0.974
Risk	0.855	0.045	0.00	0.766	0.944
Total score	0.863	0.041	0.00	0.783	0.943

Std = standard, CI = confidence interval

## Discussion

To date, there are a limited number of studies looking at the forensic outcomes of high security patients who have been discharged via medium secure care [27,28]. In this study the 72 HSPH patients had similar characteristics to those described in other MSUs e.g. [28,41-44] in that they were predominately male with extensive forensic and psychiatric histories. In a pseudo-prospective study design we examined the predictive accuracy of the HCR-20 for outcomes following transfer from high to medium secure psychiatric care. As far as we know this is the first international study to look at the HCR-20 in this way as most studies have focused on either institutional or community violence [12,16,29,33,35-37,45-48]. It is also the first to report data on the validity of this measure at predicting a broader range of outcomes following transfer to lower levels of security in the UK or elsewhere. We predicted that high scores on the Historical Clinical Risk -20 scale would be predictive of poor outcome in medium secure services. We did indeed find that the HCR-20 score was a good predictor of failed transfer. The total score ROC AUC curve was 0.86 which is much higher than the modest to moderate ROCs reported in many previous studies [9]. It is also noteworthy that it was the clinical and risk management subscales that contributed most to this effect. Studies have reported varying degrees of contribution from the dynamic subscales but the research evidence seems to suggest that the contribution of dynamic scales vary as a function of the stage of rehabilitation. In Gray's et al's [33] pseudo prospective 2 year follow up study of patients discharged from medium security to the community only the Historical and Risk scales were predictive. The clinical scales did not show notable accuracy. They suggest that the lack of predictive accuracy in their sample may reflect the clinical stability of those deemed suitable for discharge to the community as well as the differences in follow up time. Our finding that the clinical and risk items both contribute significantly to the prediction of poor outcomes fits with our previous studies in medium secure samples [16,45] and also fits with the notion that the clinical items may be more robust predictors of negative outcomes if failure is also determined by clinical issues such as lack of response to medication. There are a number of studies that have compared the post discharge outcomes of patients and using the HCR-20 with Violence Risk Appraisal Guide [49] and the Psychopathy Checklist Revised [50] or Psychopathy Checklist- Screening Version (PCL;SV.[51]) which are measures of psychopathy that have been shown to be predictive of post discharge violence [52]. In one study [53] 193 psychiatric patients were assessed using both the HCR-20 and The PCL: SV. At 2 year follow up, the AUCs for the HCR-20 ranged from 0.76-0.80 for a range of aggressive and threatening behaviors, but the PCL: SV had only moderate predictive power. Interestingly, the HCR-20 had incremental validity over and above the PCL: SV. Similar findings were noted in our previous prospective 24 week follow up study of patients discharged from medium secures and civil psychiatric settings work who had been assessed using the HCR-20, VRAG and PCL:SV[45]. Here we found that the HCR-20 and PCL:SV were better predictors of violence post discharge than the VRAG, but in the regression analyses the HCR-20 (particularly the clinical and risk scales) had incremental validity over and above the PCL:SV [45]. A Swedish retrospective study on 40 male forensic patients [37] also found that the HCR-20 was highly predictive of violent recidivism and that the clinical and risk management scales predicted recidivism much better than the historical scale. Overall, our findings seem to suggest that the HCR-20 is a useful tool in predicting those who will fail in their rehabilitation. The broader literature also suggests that it has utility in predicting post discharge recidivism (particularly violent outcomes) for both forensic and correctional samples [9]. There is a growing literature that suggests it has utility in predicting in-patient aggression and outcome [35] although the findings have been less robust as in-patient aggression may be more associated with heightened affect and active psychotic symptoms in US studies [12]. While there is now little doubt that structured risk assessment instruments outperform clinical judgment for the prediction of violent behavior and poor outcome for predominately male samples [6,11], there is relatively little data on female forensic or correctional samples. The vast majority of risk assessment studies in women have been based on psychopathy assessments [54,55] and there is limited data on the validity and utility of the HCR-20 in women [56]. Some studies looking at gender differences in the HCR-20 do not note significant differences between men and women [8,14] however, work by de Vogel & de Ruiter [57] showed that the HCR-20 total score demonstrated lower predictive accuracy for violent outcome in women compared to men. Given the observed gender differences future studies need to address this issue[15].

## Limitations

There are a number of limitations to this study including small sample size and a focus on a mainly male Caucasian cohort. Given recent reports that there are gender and ethnic differences in scores on some HCR-20 items this is an area that warrants further study [14,15,64]. Furthermore, although our cohort were fairly representative of patients detained in medium levels of security in the UK, they may not be comparable to cohorts of medium secure patients in other European and US jurisdictions where there may be greater representation of ethnic minority groups and female patients. It is also possible that the findings may not be generalisable to high security samples as this cohort had already been clinically selected as suitable for transfer to lower levels of security. In this study, we relied on clinical recording of multi-axial diagnoses, rather than standardized assessment tools. While the clinical files do record multi-axial diagnoses, it is possible that the lack of assessment using structured assessment tools may have resulted in under recording of Axis II and III pathology in particular.

## Conclusions

The findings from this study would suggest that measures such as the HCR-20 may have value in routine clinical decisions as they may assist in the assessment of those who are likely to succeed or fail on trial leaves to lower levels of security. Although the HCR-20 is increasingly being adopted into clinical practice in European forensic settings including Germany, Sweden and the Netherlands, there are relatively few UK centers outside high secure forensic facilities that use the HCR-20 as a core component of routine clinical practice. The Edenfield Centre Medium secure unit in the North of England has adopted this instrument into routine clinical practice following a series of research based validation studies to examine its utility as part of its ongoing risk assessment research program. We have shown that it is a robust predictor of post discharge outcome (readmission and self report violence) in patients discharged from our medium secure service [16]. We have also shown that the HCR-20 is one of the most robust predictors of community violence 24 weeks post discharge in patients discharged from both forensic and civil psychiatric services [45]. More recent studies by Gray and colleagues [33] confirm the validity of the HCR-20 in the prediction of violent recidivism in patients discharged from medium secure units in the UK. Several services in the United States and Europe have also published research studies supporting its reliability, validity and clinical utility across a range of levels of security as well as the community [9]. A key strength of the HCR-20 is its utility in guiding clinical judgment about risk management and it is this aspect of the instrument that has lead to its acceptance into routine clinical practice [13]. The development of the HCR-20 companion guide [10] has assisted with this process, but more work is needed to refine the role of structured risk assessment tools in clinical decision making [58]. Many studies rely on official records of reconviction as an outcome measure. We suggest that there are limitations in the use of reconviction data as a proxy measure of success in assessing the efficacy of forensic services [59,60] including the fact that there may be bias in the prosecution of psychiatric patients which limits the accuracy of this data in assessing and comparing outcomes [61,62]. This however remains one of the most cited performance indicators. In recent years, there has been a move away from reliance on criminal outcomes alone and recent work suggests alternative measures such as readmission and collateral and self reported criminality may be useful indicators of outcomes [16,45]. Further studies are needed to track and monitor the mental health and criminal outcomes of patients discharged from high and lower levels of security and to compare the outcomes of patients who are discharged to the community and followed up using an integrated, as opposed to a parallel, model of aftercare [62].

## Competing interests

The authors declare that they have no competing interests.

## Authors' contributions

MD conceived of the study, and participated in its design and coordination and drafted the manuscript. RB carried out the field work, assisted in data analysis and assisted in drafting the manuscript. All authors read and approved the final manuscript.

## Pre-publication history

The pre-publication history for this paper can be accessed here:

http://www.biomedcentral.com/1471-244X/10/76/prepub
